# A retrospective analysis of the change in anti-malarial treatment policy: Peru

**DOI:** 10.1186/1475-2875-8-85

**Published:** 2009-04-28

**Authors:** Holly Ann Williams, Arlene Vincent-Mark, Yenni Herrera, O Jaime Chang

**Affiliations:** 1 Malaria Branch, Centers for Disease Control and Prevention (CDC), Mail Stop F-60, 4770 Buford Hwy NE, Atlanta, Georgia 30341, USA; 2 Division of Injury Response, CDC, USA; 3 Ministerio du Salud, Jesús Maria – Lima, Peru; 4 United States Agency for International Development (USAID), Surco, Lima, Peru; 5 International Emergency and Refugee Health Branch, Centers for Disease Control and Prevention (CDC), Mail Stop F-60, 4770 Buford Hwy NE, Atlanta, Georgia 30341, USA

## Abstract

**Background:**

National malaria control programmes must deal with the complex process of changing national malaria treatment guidelines, often without guidance on the process of change. Selecting a replacement drug is only one issue in this process. There is a paucity of literature describing successful malaria treatment policy changes to help guide control programs through this process.

**Objectives:**

To understand the wider context in which national malaria treatment guidelines were formulated in a specific country (Peru).

**Methods:**

Using qualitative methods (individual and focus group interviews, stakeholder analysis and a review of documents), a retrospective analysis of the process of change in Peru's anti-malarial treatment policy from the early 1990's to 2003 was completed.

**Results:**

The decision to change Peru's policies resulted from increasing levels of anti-malarial drug resistance, as well as complaints from providers that the drugs were no longer working. The context of the change occurred in a time in which Peru was changing national governments, which created extreme challenges in moving the change process forward. Peru utilized a number of key strategies successfully to ensure that policy change would occur. This included a) having the process directed by a group who shared a common interest in malaria and who had long-established social and professional networks among themselves, b) engaging in collaborative teamwork among nationals and between nationals and international collaborators, c) respect for and inclusion of district-level staff in all phases of the process, d) reliance on high levels of technical and scientific knowledge, e) use of standardized protocols to collect data, and f) transparency.

**Conclusion:**

Although not perfectly or fully implemented by 2003, the change in malaria treatment policy in Peru occurred very quickly, as compared to other countries. They identified a problem, collected the data necessary to justify the change, utilized political will to their favor, approved the policy, and moved to improve malaria control in their country. As such, they offer an excellent example for other countries as they contemplate or embark on policy changes.

## Background

Throughout malaria-endemic areas, national malaria control programmes must deal with the challenges of changing malaria treatment policies in response to unacceptably high levels of drug resistance to previously used anti-malarial drugs, such as sulphadoxine-pyrimethamine (SP). The current 'gold standard' for treatment of uncomplicated *P. falciparum *malaria is use of artemisinin-based combination therapy (ACT)[1]. While global guidelines offer information on the best drugs to use, there have been only a few studies published about the *process of change *in countries in which policy changes have been made [2-9]. However, some of these studies specifically focus on drug efficacy data as the primary issue in changing malaria treatment policies [6,10], as opposed to the social and political components of policy change.

Selecting the most appropriate and efficacious drug is only one of many facets of changing malaria treatment policy. Effective policy change is a long, involved process that extends for months to years and requires input from a multitude of stakeholders, both public and private[2,7,11]. Steps of the process include: a) being aware that a change is needed, b) verifying data to ensure that a change is required, c) presenting data in language that is easily understood by all involved in the policy cycle, d) advocating for the proposed change, e) fostering agreement among all stakeholders that a change is required, f) identifying policy options and selecting the most appropriate, g) agreeing on replacement drug/s, h) developing consensus on timing for the change, h) developing all policy documents, i) completing all preparatory steps for implementation, j) implementing new policy, k) monitoring and evaluating the change, and l) planning for next policy cycle [2]. Some of these steps may occur simultaneously.

During the process, it is very important to also pay attention to economic, political, legal/regulatory, socio-behavioural, environmental, and other contextual factors that impact the process of change [2,12]. Competition for scarce resources among various national sectors; lack of adequate planning; national and regional political agendas; cost, efficacy, availability, safety and acceptability of the replacement drug/s; ineffective communication and limited trust between scientists and policy makers; status of the public health care system in general; legal and regulatory statutes; fluidity of national borders; degree of decentralization; local epidemiological context; and vested interests of stakeholders (particularly the pharmaceutical industry) are examples of factors that can significantly influence the process of drug policy formation and implementation.

In response to growing levels of anti-malarial drug resistance to chloroquine (CQ), in 1999, Peru decided to change its national malaria treatment policy. Information pertaining to the proposed change was published in a document entitled "Politicas Nacionals"[13]. In 2001, a site-specific approach to malaria treatment policy was formally approved. For the Macro Región Norte (Northern Coast), the Ministry of Health (MoH) selected the combination therapy of SP and artesunate (AS) as first-line therapy. For first-line treatment in Macro Región Amazónica (Amazon Region), the MoH chose mefloquine (MQ) and AS. With the assistance of the Peruvian MOH and the United States Agency for International Development (USAID) (through the Amazon Malaria Initiative [AMI]), the Centers for Disease Control and Prevention (CDC) initiated a retrospective analysis of the change in anti-malarial treatment policy in Peru.

This paper offers a historical review of the process from the early 1990's to early 2003, identifies factors that assisted or hindered the process of change, summarizes 'lessons learnt,' and offers recommendations for subsequent changes as derived from the shared perspectives of the various stakeholders. While most of the paper focuses on the process surrounding the process of gaining consensus for change, the drafting of the new policy and the selection of replacement drugs, reference is also made to issues regarding implementation, which can be thought of as the second phase of change. Stakeholder quotes derived during the interviews are used to illustrate concepts. The process is primarily describing the events at the national (central) level, with some discussion of events at the two regional areas (Amazon Region and Northern Coast).

## Methods

The study was reviewed by the Institutional Review Board at the Centers for Disease Control and Prevention and found to be exempt. Individual interviews and focus group discussions (FGDs) were held with key stakeholders who had been involved in the process of changing the policy, primarily at the central level. The sampling frame was created by two methods: a purposive sample was generated based on names of stakeholders that were involved and/or had information about the process of Peru's policy change (this initial list was compiled by Peruvian colleagues), and the snowball sampling technique, in which additional persons are identified and asked to participate based on recommendations given by persons already interviewed. This case study also used stakeholder analysis techniques to ensure that relevant parties were included in the sampling frame and to understand the influences stakeholders had on the policy process[14,15].

All interviews used a broad, open-ended approach to gather data. The questions were focused on describing the *process *of change, including describing the wider socio-political context in which the change occurred. Following the initial question asked: "*can you please describe the process by which policy change occurred in Peru?" *other questions were generated. Methods to obtain data also included a participatory technique of developing a timeline of key events (developed by consensus among stakeholders) and reviewing documents pertaining to the change in policy. The research team wrote extensive field notes during the interviews and compared their notes after each interview to ensure accuracy and completeness. Verbatim quotes are used in this paper to illustrate concepts or points of view.

Content analysis was used to analyse the qualitative data. Verbatim notes from the interviews were coded, from which themes and categories were identified. Individual interview data were compared to focus group findings. During one focus group discussion, all participants were asked to generate a timeline of key events. Using the individual timelines, a "master" timeline was developed. Any specific individual data that did not match with the master data were discussed with the entire group and either added to the master timeline or a reason was given for why it was not considered a key event. All participants in the focus group consensually agreed upon the master time line.

The researchers reviewed copies of all documents that related to the process of change to verify dates/events that had been described in the interviews. Lastly, a draft of the report and findings was presented to some of the key stakeholders. Stakeholders raised issues for clarification and mutually agreed upon the findings as presented by the researcher, with minor edits.

## Findings

### Sample

Thirteen individual and four focus group discussions were conducted during two visits to Lima, Peru (February and July, 2003). A 14^th ^individual interview was conducted as a phone interview in Spanish, with the transcript of the call translated into English, as the participant had not been available during the visits to Peru. Most interviews were done in a combination of Spanish and English, with translation provided as needed.

Individual interview participants were from various organizations: Ministry of Health (MoH) (n = 4), private consultants (n = 2, both of whom were former MoH staff), donor agencies (n = 3, all of whom were prior MoH staff), international technical/policy agency (n = 1, prior MoH staff), a non-governmental organization (NGO) (n = 1, also part of MoH staff), media representatives (n = 2) and a pharmaceutical manufacturing representative (n = 1).

Focus group discussions represented key stakeholders, including participants from the MoH staff, World Health Organization regional staff, Department level (DISA) staff, NGO and donor representatives. Some participants were involved in both individual and focus group discussions. Triangulating the data by obtaining information by both individual and FGDs interviews served as a way to clarify perceptions, verify the data in general, and obtain consensus on the key events and the timing of those events. One focus group specifically focused on the Northern Coast provincial (DISA) level, as local DISA staff were in Lima during the time of the study. For the Amazon Region, specific questions were asked of various participants who had previously worked in that area during the time of the change, particularly those who had assisted with building local capacity as a result of the process of change.

### Historical narrative

The process of changing the Peruvian anti-malarial treatment policy evolved over numerous years and involved a host of stakeholders (Additional file 1). In addition to changing patterns in drug efficacy, environmental and political influences affected the process. The following historical narrative reflects what the participants in this retrospective analysis perceived as the most important events and factors that influenced the change in Peru's anti-malarial treatment policy.

Unlike most African countries, historically, Peru's malaria problem was primarily focused on non-falciparum malaria (before 1990, *Plasmodium falciparum *cases accounted for less than 1% of all cases). In 1990, the number of cases of *P. falciparum *began to increase. At that time, first-line treatment for uncomplicated malaria was CQ, with quinine (QN) used to treat the few failures that had been seen.

In the early 1990's, no one was alarmed even though, as early as 1991, clinicians were noticing that CQ had stopped working in some parts of Loreto. As a focus group participant described:

"Malaria resistance wasn't perceived as a problem. We assumed drugs would work fine for a long time. The process begins when there is sensitization to a potential problem, not when you have efficacy results."

At this point in time, there were no widespread standardized efforts to document the perceived changes in efficacy, although a small study was completed that documented the increased levels of resistance[16]. Efficacy data were being collected using non-standardized efficacy trials. In the Amazon Region, a decision was made at the DISA level to use SP as the first line, in spite of it being the second-line drug in the official policy. Data were presented to the central level and the Director General (DG) made a visit to the region. Although there were inconsistencies in the data from the DISA and central levels, the DG approved use of SP. This decision was then revoked at the central level, with instructions given to regional directors to return to using CQ as first line therapy. Local officials felt that this change was a big mistake and would only make resistance worse.

A regional malaria meeting was held in Venezuela during 1994 that emphasized themes from the WHO Global Strategy for Malaria: detection, diagnosis, and treatment[1]. However, due to inadequate resources, Peru directed its control efforts toward treatment and not prevention.

In 1994, an operational monitoring system for drug resistance was started by the Peruvian National Malaria Control Programme (NMCP). As part of this, health services began to offer directly observed therapy for malaria, and started a 28-day follow up for all patients diagnosed with malaria (data from this monitoring programme was referred to as 'cohort' data) [10]. Around the same time, the traditionally vertical NMCP became integrated within the general health services[10]. Whether this shift away from vertical programming was beneficial or not was debated among the interviewed stakeholders. Those who supported the change felt that it focused the problem of malaria more directly at the health care services level, meaning that prescribers were more acutely aware of malaria and could more easily see trends in drug resistance. Those opposed to the change perceived that 'malaria control' was only limited to case management and ignored prevention. They also felt that malaria technical expertise was lacking in the general health services and worried that critical areas, such as vector control, would be ignored.

Clinician reports of increasing CQ failures began to emerge from two areas: the northern coastal plain area and the Amazon region. 'Cohort data' indicated failure rates of up to 30% but the data were not perceived as reliable or rigorous and were treated with skepticism due to problems of enrollment, incomplete coverage and failure to use standardized protocols. By 1996, there was a steep increase in the overall number of malaria cases, with the proportion of *P. falciparum *increasing. Malaria cases increased four-fold in Peru from 1992–97 and 50-fold in the Amazon area of the Loreto Department[17]. By the end of 1997 and during early 1998, the weather phenomenon, El Niño, produced increased rain and flooding, followed by an outbreak of malaria on the northern coastal plain[18]. Due to the concern about increasing levels of anti-malarial drug resistance, in 1996, SP became the first-line treatment for uncomplicated malaria in Loreto (Central Amazon) and the Brazilian border area of the Eastern Amazon.

The increase in malaria cases in the Amazon Region in 1997 resulted in five Director Generals (DG) visiting the Loreto area in order to develop a plan of action. Unfortunately, most of the DGs left before decisions were reached on how to proceed. However, in the eastern Amazon region, SP was replaced with a 7-day course of QN and tetracycline in 1997[10].

A former NMCP staff member noted that they [NMCP] had heard about resistance in other countries and had even planned ahead for the eventual possibility of the need to change anti-malarials. In reality, few people were ready for the speed in which events occurred.

"We thought and planned ahead, had it in mind that we might have to switch to other anti-malarials. However, we did not think that the need to change would come so quickly. This caused us to be about two years behind in starting drug efficacy studies."

A consequence of this was that there was prominent media coverage focused on the increasing number of malaria cases and the desire for insecticide spraying, as this had been an effective strategy when used previously. The media coverage sharpened the awareness of the community, the NMCP, and physicians within the MoH about the resurgence in malaria. The press accused the MoH of not doing enough to prevent and control the problem. The response by the MoH was directed toward placing the responsibility back at the provincial level, rather than the central level, although no additional resources were released to the DISA level to help with the epidemic situation. Ministry of Health staff felt that the Northern Coast was well prepared to deal with El Niño. However, others felt that El Niño overwhelmed the capacity of the MoH at that time, although the situation improved with time. During this time, the MoH perceived their relationship with the media as positive and 'open.' These sentiments stood in stark contrast to others who stated that the MoH reacted defensively to the criticisms leveled and failed to provide adequate figures that described the situation.

During this period of time, events at both the global and regional level occurred that would impact the situation in Peru. At the global level, the World Health Organization (WHO) published a standard protocol for conducting malaria drug efficacy trials. Use of this protocol would later assist in improving efficacy data collection. At the regional level, WHO/Pan-American Health Organization (PAHO) conducted an external evaluation of the Peruvian programme, releasing a statement saying that the malaria control strategies employed by the MoH were appropriate in response to El Niño and should be supported by the Ministry of Health. Provided as a press release to all the major newspapers, the statement was seen as an international endorsement of the efforts of the Ministry.

As well, stakeholder interests in anti-malarial drug resistance and emerging infectious diseases began to meld[10]. Collaborative relationships began among different agencies: the Peruvian Instituto Nacional de Salud (INS) [the institution within the Peruvian Ministry of Health that is responsible for public health research and training], USAID, CDC, United States Naval Medical Research Institute Detachment (NAMRID), and the Loreto DISA.

In March 1998, PAHO held a meeting in Manaus, Brazil to discuss revision of the WHO standardized drug efficacy protocol, in order to make it more appropriate for use in the Amazon Region. The meeting was attended by a variety of people including INS representatives, staff from the NMCP, faculty from the School of Public Health and CDC. After returning from the meeting, the group was able to convince the director of the INS that the issue was important. A decision was also made to adopt 'lot quality assurance' (LQA) sampling for their cohort data, which was seen as a critical strategy for standardized sampling.

Following the PAHO meeting, a September meeting was held in Lima to further discuss standardized efficacy protocols. At this meeting, there was tension between university researchers who felt that this type of research needed to be academically based (with patients hospitalized for follow-up) versus using operational research from within the control program, assisted by the national research institution. Agreement was reached on using the WHO standardized drug efficacy protocols, particularly after recognition of the usefulness of lot sampling. As a result, by using LQA, the researchers decreased their variability across the samples; thus, improving their data.

In 1998, a key event occurred that would prove to be beneficial to the process of changing treatment guidelines. The "VIGIA Project," which had the political support of the Minister of Health and financial support from USAID, was conceived as a five-year project to strengthen national and local capacity to identify, control and prevent emerging and re-emerging infectious diseases more effectively. An important feature of this project was that the VIGIA implementing partner was the Peruvian MoH, which then designated a National Team responsible for activities that would report at the Vice-Ministerial level. The Project worked closely with other major MoH divisions, as well as outside partners. As such, it was able to play a critical role in promoting collaboration among partners and stakeholders throughout the policy process in Peru. The membership of the project team included colleagues that had worked together for many years in malaria. The strength of these social and professional networks enhanced the Project's ability to interact effectively with the malaria control programme.

During this time, a protocol for research to inform the change in treatment guideline process began to be developed by staff from the INS. It soon became clear that the various involved agencies held differing perspectives as to how the research should be done and who should be responsible for the research. As INS was developing the protocol, they felt as though they should be the ones to conduct the research, although they lacked the funds to do so. The NMCP had the mandate to do the research but had some trepidation about moving forward for fear of the results. There was concern that if the findings indicated a high level of resistance, there would be criticism leveled at them that they had not done their job properly. They also lacked funding for research and were not prepared to change the drugs should the results require that action. Despite the situation of having multiple organizations involved that, at times, had different objectives, these stakeholders were able to align their interests and join efforts in order to develop research protocols that would help in moving the development of the new anti-malarial drug policy forward.

As well, discussions ensued over the use of the new standardized WHO drug efficacy protocol versus using an older one. Ultimately, the newer one was adopted and implemented. Capacity building started with VIGIA providing partial funding for research and training and one researcher from Peru received funding from CDC to travel to Atlanta for further training. Through an epidemiology training program, an additional 39 people acquired more epidemiology skills. As well, during this time, a national network of laboratories was promoted through the National Laboratory.

Throughout this time, staff from the DISAs were included in regularly held Task Force meetings in both Lima and Iquitos (largest city in the Peruvian Amazon area). By including DISA staff during all phases of the process (from data collection through analysis and dissemination), they built local capacity. As one MoH official noted:

"They became part of the solution if there were problems."

Efficacy trials were conducted by INS in the Iquitos area on CQ and SP, in collaboration with CDC. Health professionals began to voice concerns about increased anti-malarial drug resistance.

'Cohort studies' continued and the DISA data given to the NMCP were indicating growing levels of resistance. In July 1998, a team from the INS presented their preliminary results from the efficacy trials to their partners and the scientific community, with results demonstrating more resistance to both CQ and SP. By September 1998, a decision was made to replace SP with QN and tetracycline, although this never was implemented until early 2000[10].

In 1999, results from standardized efficacy studies were demonstrating resistance levels of greater than 30% to CQ but not SP in Northern Coast areas. The results were important in demonstrating not only high resistance levels, but also that there were differences between the two eco-systems, thus requiring a site-specific policy. In June of 1999, SP was chosen for the first-line therapy for *P. faciparum *infections in the areas of the Northern Coast.

Politically, during this time, there was a key stakeholder within the Ministry who had direct access to the Minister. Having this person directly linked to the Minister allowed the involved stakeholders to informally channel messages about the need for malaria treatment policy change to a top political figure.

During 1999, the first draft document discussing the need for change, 'Politicas Nacionals,' was drafted and circulated after standardized efficacy results were discussed with stakeholders[13]. This document only reflected preliminary decisions, noting that there would be a change in anti-malarial treatment policy, without specifying which drugs would be used as the replacement drugs. It outlined necessary steps for the change in policy, urged drug efficacy testing of new drugs, and discussed general drug requirements, including issues like drug safety, need for simplicity, acceptance and compliance. The MoH officially endorsed this policy protocol on October 15^th^, 1999.

Prior to official sanctioning, to promote advocacy for the change, the Peruvians used a strategy not seen in the other countries. They held an open meeting in the capital city of Lima in August 1999, inviting all stakeholders to discuss the draft protocol. They also presented drug efficacy results from seven sentinel sites. Rather than using the external technical experts who had assisted with the studies to present the data, the provincial level staff presented the findings. This increased the DISA-level sense of ownership of the process. Simple graphs were presented to display the increasing levels of malaria-related morbidity and mortality and technical jargon was removed so that non-technical people could understand the messages.

In conjunction with the open meeting described above, they conducted an open evening seminar, directed toward the public, which had been publicized through a quarter page advertisement in the major newspapers (resulting in attendance of about 300 people). At this forum, the topic of discussion first centered on the problem of resistance globally, moved to the status of resistance within Latin America and, finally, narrowed to the problems seen in Peru. To support their arguments for possibly introducing combination therapy, they discussed experiences from SE Asia as a reference point. External partners presented this information. Using technical experts with international experience in malaria strengthened and supported the credibility of what the national experts were saying.

As well, those involved in the change recognized the need for supplemental economic data to support the drug efficacy results. They conducted an analysis of the economic burden of the problem and presented findings as the costs to the nation as a whole, rather than solely as costs to the MoH. They included factors such as loss of production from work absences and indirect costs related to malaria.

Efficacy testing on anti-malarial combination therapies continued until June 2000, when it was decided that sufficient data had been gathered to document levels of efficacy and safety. A decision was made to initiate a formal system for monitoring resistance, which would incorporate surveillance data from the cohort data and use of sentinel sites. The official policy, outlining the replacement drugs for the site-specific treatment guidelines, was announced in June 2000. The team involved in the process gave the following recommendations for combination therapy to be used as temporary measures against uncomplicated *P. falciparum*: a) Northern Coast: SP/AS and b) Amazon Region: QN and tetracycline (or MQ/AS). Pregnant women would be treated with QN and clindamycin and children under eight would use the combination of QN and clindamycin. Additional drug efficacy studies would be conducted and the replacement drugs would be selected based on those data.

Letters were sent out to the provincial levels, explaining that there had been a national decision to change malaria treatment policy, although no date was set for when drugs might reach the periphery. Initially the provincial level voiced resistance to the protocol, noting that a major constraint was insufficient funds to purchase new drugs. Their responses reflected the political orientation of the country at that time. The Fujimori government was in the last months of power and there was pressure from the government for the provincial levels to decrease expenditures. Officials were being jailed for over-expenditures; thus, there was reticence to agreeing to a measure that would increase costs. A call for tender proposals for anti-malarials was issued to national suppliers but the process was slow due to continued fear of reprisals for spending funds. However, the new anti-malarials were incorporated into the national formulary and replacement drug purchasing began with the out-going government.

In 2001, using cascade training, the national team trained physicians and nurses from six regions on the new treatment policy, followed by the DISA-level staff training district-level workers, with some support from the Ministry. Although the focus was on the Northern Coast and Amazon regions, other areas affected by labor migration were also included. The budget for training was not clearly defined and available, which delayed implementation. Historically, in the previous vertical programme, all malaria-related training was assumed and paid for by the NMCP. With decentralization, DISA-level budgets were not adjusted to account for the necessary training. Luckily, there was some supplemental funding from VIGIA available for training.

Although the training materials were developed at central level, the DISAs distributed the materials. It was noted that there was limited diffusion of training materials at the local level, particularly in the Amazon region, where transport of materials by river proved to be a major challenge. There was some criticism of the way training was conducted, with some stakeholders noting that the MoH did not have the capacity to provide adequate training; thus, the universities should have been more involved. To counter this, other stakeholders noted that the universities had been invited on numerous occasions to participate and chose not to become engaged in the process. The general consensus was that training and the production of Information, Education and Communication (IEC) materials needed to be improved.

There was also a strong recognition that drug resistance was a regional problem, particularly due to the high rates of cross-border migration that occurs between Peru and its neighbouring countries of Ecuador, Columbia, Brazil and Bolivia. As part of regional efforts to standardize and coordinate drug efficacy testing, the Peruvian MoH went to Bolivia to train 60 physicians in drug efficacy monitoring in 2001.

While focusing on the importance of regional approaches, the key stakeholders also never lost sight of the need for country-level data. As a former Director General noted:

"Theoretically, you can know geographical distributions, epidemiological and other influences from other countries, but the population wants it specific to their own country. People are wary and do not want an experiment from another country."

By this time, the transitional government had been formed and elections were held in July 2001. At the very end of the transitional government, the new officials in the Ministry of Health agreed to the change in policy and on August 7^th^, 2001, the Director General of "People's Health" department signed a revised national malaria treatment policy, which outlined the first-, second- and third-line anti-malarials to be used for the treatment of uncomplicated *P. falciparum *malaria[19]. The revised guidelines followed the previous recommendations for the site-specific combination therapies. The MoH announced the change in the major newspapers a day after the official signing ceremony.

Although training had started, drugs were not yet available. The transition government had not wanted to purchase the new anti-malarials, as the process initiated by the Fujimori government was perceived as corrupt. Purchasing drugs started again in September/October 2001, although it was a very slow process. By late 2001, the first batches of the new drugs were brought to the critical areas and the new treatment policy was officially implemented in November 2001[10]. 'Cohort studies' continued as a matter of routine operational business by the control programme.

**Figure 1:**
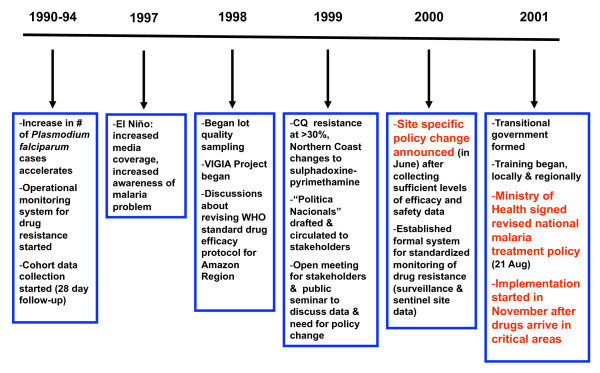
**Key events in changing Peru's anti-malarial drug policy**.

Due to the loss of staff resulting from a change in the government, re-training had to occur, starting in August 2002. Regional-level teams conducted the trainings, with support from the central programme. Evaluation of the implementations was initially planned for 2002 but, since implementation had stalled, it was rescheduled for later in 2003 (Figure 1).

### Political context

The wider political context affected the process of policy change in Peru. With each change in government, officials changed within the Ministry of Health. Initially, with the transitional government, there was extremely close scrutiny of all activities, and fear of using funds for procurement of drugs and supplies was widespread. This resulted in procurements being halted, affecting not only anti-malarials, but also vaccines and insecticides. As mentioned earlier, the tender for anti-malarials was issued in 2001, but the process was slow. By the time procurement occurred in 2002, a crisis point had been reached, as supplies fell below the critical level. One focus group described this period as:

"Political will was there, but there were no drugs!"

There were strategies in place whereby PAHO could have assisted with procurement, but the Peruvian government made no such request. Some provincial departments were able to purchase drugs from their own budgets, but this was not consistently done. As a donor representative described:

"There was paralysis in the entire Ministry and it was hard to deal with."

Generally, procurement was not on schedule during the transitional period and specific items were not included in the budget. By December 2002, the drugs had arrived to the level of the health services and coverage was good by January 2003. As of February 2003, there were sufficient supplies to last until June 2003. As well, an agreement was reached to allow PAHO to purchase drugs, effective in 2004.

#### Loss of capacity

The changes in government clearly had a negative impact on the level of trained personnel available and continuity within the control programme. It was felt that the new governments perceived anyone who had worked in the Fujimori government as being corrupt, resulting in many technical staff members losing from their positions. Each time a new government came into power, the MoH endured a tremendous loss of human resources that had solid technical experience in public health. For example, during 2002, one endemic region lost 25 out of 37 workers assigned to malaria control. As a donor described the impact of staff loss:

"The problem is that in many places the staff is not good enough to make technical or operational decisions."

Another former Director General in the MoH noted:

"You cannot work with peace in your mind if you don't know that you'll have a job."

Many health staff that had been trained in the initial cascade training lost their jobs. This increased the complexity of implementation as new workers needed to be trained, which was particularly difficult in the remote Amazon region.

### Identification of opportunities

Recognition that local capacity could be developed to assist in the process was another key factor in moving the process forward. Not only was local capacity developed in terms of monitoring drug efficacy, DISA level staff gained experience in dealing directly with the central level, which resulted in the central level becoming more receptive to a shift of power to the local level.

They also recognized that it was very useful to identify and capitalize on environmental or political events occurring that might provide additional support for the change in policy. For example, El Niño was an event that could not have been planned in advance to coincide with the need to change policy, yet it served to stimulate interest in their cause. As one INS staff member stated:

"You need to seize the moment and opportunities to make things happen."

In addition to the local events, global discourse about the threat of increasing resistance helped to sensitize people to the immediate problem Peru was facing, as well as what it meant regionally. PAHO used the example of events in Africa as an impetus for change, which lent support to the process in Peru.

As well, the NMCP recognized that the Health Commission in Parliament was very interested in malaria, as physicians from the Amazon region were members of this commission. As these members had a vested interest in improving malaria care in their home areas, the NMCP utilized this opportunity to obtain support for the needed policy changes by providing them malaria information.

Other opportunities that assisted the process included funding from VIGIA and NAMRID. Although the objectives of NAMRID were not necessarily consistent with the NMCP or the INS, their common research interests provided additional funding.

### Challenges to overcome

Throughout the process, stakeholders were consistent in describing the challenges that they faced. Initially, there was internal reluctance within the NMCP to recognize that the time had come when it was necessary to change anti-malarials.

Frequent changes in the ruling government created serious problems that affected the process of change. These included: a) loss of historical memory that made the process fragmented, b) limited human resources due to political changes, with simultaneous loss of both policy makers and technical staff, c) difficulties with malaria-specific decision-making due to loss of technical expertise, d) unstable health systems with insufficient number of workers dedicated to malaria, e) heightened anxieties from health care workers about career stability, and f) uncertainties about budget allocations and the ability to fund the needed changes secondary to fears of political reprisals. As one MoH staff member commented:

"Once the budget was defined, there was no clear definition of what it implied. There was an idea that it would cost more but how much more was not clear."

### Lessons learned

Stakeholders were asked to reflect on the entire process of malaria policy change in Peru in order to identify key lessons learned. The following list, not prioritized in any order, represents what the stakeholders felt were the most salient 'lessons learned':

a) Engage all stakeholders at the beginning of the process.

b) Promote change through the use of rigorous scientific data.

c) Orient scientific data toward malaria control needs, rather than the needs/interests of the researchers.

d) Make use of existing opportunities or create opportunities to put malaria on the political agenda.

e) Demonstrate to policy makers that it makes good economic sense to change policy: show the effect of not doing something. Focus discussions regarding cost-benefits to the general population, rather than only to the Ministry of Health.

f) Present data with the support of international technical experts, which strengthens the credibility of national researchers.

g) Always present solutions when presenting problems to policy makers.

h) Develop an implementation plan with someone higher placed in the MoH than the NMCP, who has direct access to policy makers.

i) Ensure all systems and supplies are in place before attempting implementation so that scarce resources are not wasted.

j) Develop policy in a collaborative manner and share experiences with neighboring countries.

In additional to identifying "lessons learned," stakeholders were asked what advice they could offer others who are starting the daunting process of changing their anti-malarial drug policies. The following recommendations were offered:

a) Use an integrated team approach and recognize from the beginning that your work will lead to a change in drug policy – identify your end goals.

b) Conduct periodic monitoring for drug resistance and develop good surveillance systems.

c) Obtain assurance from the administrative side that they will work in tandem with the technical partners, particularly in the context of decentralization.

d) Evaluate policies constantly to identify existing problems and to look ahead.

e) In the context of decentralization, provide technical support to district/regional levels as needed.

f) Promote regional approaches to policy formulation.

g) Focus donors on the goals of the country:

"In order to have success, individual donors should have one objective, which is to help Peru, not their own objectives!" (quote from international technical agency)

h) Utilize the principles of strategic planning, particularly in the area of drug management (procuring appropriate levels of drugs in a timely manner and distribution of the drugs peripherally).

## Discussion

In spite of the political turmoil and environmental changes, the Peruvians utilized a number of strategies successfully to ensure that policy change would occur. The process was driven by a group of individuals who possessed a common interest in malaria and the political will to move the process forward in a fairly uniform fashion. Although the ruling government experienced major transitions throughout this process, the key stakeholders provided continuity and institutional memory in the face of a government that could not offer such stability.

Key elements included: a) teamwork among individuals from diverse agencies, b) trust and linkages among team members from previous years of working together as colleagues, c) respect for and inclusion of regional and district level health care workers in a decision situated at the central level, d) demonstrated high levels of technical and scientific knowledge, e) use of globally sanctioned standardized protocols for data collection, and f) transparency in the process. As with other countries that have recently modified their malaria treatment policies[9], Peru used the strength of collaborative relationships among stakeholders to their benefit. Those networks were, in part, driven by both current and previous NMCP staff members.

They capitalized on movement of key individuals upward within the Ministry of Health, who could be strong advocates for the change. They pushed the proposed policy changes forward through using political pressure from a former Head of the NMCP, who was promoted to the level of General Directorate. The process was strengthened also through collaborative relationships with global partners. Lastly, they stressed the idea that the process was a "win win" situation, resulting in effective drugs being dispensed and local and national capacity strengthened (Additional file 2).

The strategies employed by the Peruvians mirrored factors identified in the literature that promote improved take-up of research by policy makers and enhance rational policy formulation. These include: a) exchange of information on a regular basis between scientists and policy makers, b) ensuring that policies are informed by sound scientific information, c) early and continuous engagement of stakeholders at all levels (from national to peripheral), d) timeliness and local relevance of the research, e) identification of key networks that may influence decision making, and f) understanding the complicated context in which policy formulation and implementation resides, including both the process of decision making and the formal/informal channels of power[2,7-9,20-27].

Determining the best, most sustainable and practical policy is only one early step in the process of drug policy change. Implementation follows the establishment of policy and can be a daunting task for NMCPs. Although issues related to implementation were only touched on briefly in this paper, the challenges identified by the Peruvians (such as problems with financing and procurement of the replacement drugs, loss of staff, etc) were not unique to Peru. A recent paper from Kenya noted that it took over 32 months from announcing the change in policy to completing early implementation[8]. Malawi also experienced significant delays during the times they have changed their malaria treatment policies, with implementation lags of two to three years[9].

An interesting, unexpected benefit of conducting this research and engaging key stakeholders in the process was that those most involved in the process had not realized, at the time, that what they were doing was essentially changing policy. This revelation came to them only during the retrospective review of their work. As they explained:

"We didn't think of this as policy. It was a decision that had to be made and we worked on it. You see, policy is normally associated with politicians, often seen as troublemakers!"

"We do these things as routine behaviors and we do not think about it. Yet, your questions [referring to the lead author who was assisting the control program to document their changes in policy] make us ask ourselves what we did. Now we see the need to document and write this all down."

## Limitations

There were several limitations to this retrospective study. First, due to a variety of factors, field visits were not possible to the Northern Coast or the Amazon Region, although a lengthy FGD was held in Lima with DISA representatives from the Loreto area. Thus, perceptions from the DISA level are limited. The study was limited to the perspective from the major stakeholders and did not include perceptions from the community. Another area in which there was limited discussion relates to the process of decentralization. During the time of data collection, decentralization was in process, with specific Ministries being decentralized at different times. The MoH was to be one of the last to be decentralized, due to its size and complexity. Thus, there are only scattered references to the effect of decentralization on the process.

The analysis was based on a retrospective recall of events, which could have produced recall bias. To account for this, data triangulation was used, as it corrects for any individual lapse by providing a collective memory that has internal consistency.

## Conclusion

Although not perfectly nor fully implemented as of March 2003, the change in malaria treatment policy in Peru occurred very quickly, as compared to other countries. While Peru demonstrated some unique approaches to the process, they clearly shared common features noted in policy changes completed in African countries[2,8,9]. Peru was a good example of how policy reform often results from political negotiation among the stakeholders[28].

Peru offers an excellent example for other countries to follow as they contemplate their next process of anti-malarial policy change. As best said by a former staff member of the INS when discussing future policy changes:

"You cannot be afraid of challenges. On the contrary, you must look for challenges. Demonstrate and validate [through rigorous scientific evidence] your proposal. This is the best way to convince even your enemies. Make a political decision and take the power to make the change!"

Peru identified a problem, collected the data needed to justify the change, utilized political will to their advantage, gained the approval for a policy change and moved ahead to improve malaria control in their country.

**Human Subjects Protection**: The study was reviewed by the Centers for Disease Control and Prevention's ethical review board and classified as exempt.

**The findings and conclusions in this report are those of the authors and do not necessarily represent the official position of the Centers for Disease Control and Prevention**.

## Competing interests

The authors declare that they have no competing interests.

## Authors' contributions

HAW (Principle Investigator, design of study, data collection and analysis, overall project supervision, development and review of manuscript; AVM (Co-investigator, data collection and analysis, review of manuscript; YH (data collection and review of manuscript); and OJC (data collection and review of manuscript).

## Supplementary Material

Additional file 1 **Matrix of Stakeholders' Interests, Impact, Influence and Roles**. The table describes the affiliations and roles of the major stakeholders, coded to indicate levels of interest, impact and influence. Click here for file

Additional file 2 **Factors Impacting the Process of Changing National Anti-malarial Drug Policy in Peru**. Factors that impacted the change in national anti-malarial drug policy are categorized by type and directionality (positive or negative). Click here for file
