# Supplementary material for: A retrospective analysis of the change in anti-malarial treatment policy: Peru
Source: Malar J. 2009 Apr 28;8:85. doi: 10.1186/1475-2875-8-85 (PMC2684118; doi:10.1186/1475-2875-8-85)
Supplement: Additional file 1 — Matrix of Stakeholders' Interests, Impact, Influence and Roles. The table describes the affiliations and roles of the major stakeholders, coded to indicate levels of interest, impact and influence. [file 1475-2875-8-85-S1.doc]

**Additional file 1: Matrix of Stakeholders’ Interests, Impact, Influence and Roles**

| **Analysis Definitions** | |
| --- | --- |
| **Interest** | The level of *interest* that specific individuals, organizations and groups  had in drug policy change process in Peru in the period under analysis. |
| **Impact** | The level of *impact* or *effect* that the drug policy change in Peru would  have had on specific individuals, groups, organizations in the period under analysis. |
| **Influence** | The level of *influence* on the drug policy change process in Peru that  specific individuals, groups, organizations had through their provision of  resources (financial and/or technical support, scientific resources), and  ability to generate community pressure. |

**List of Acronyms**

**CDC** Centers for Disease Control and Prevention

**DISA** Peruvian Ministry of Health staff at the Department level*
**GOP** Government of Peru

**INS** Instituto Nacional de Salud

**NAMRID** U.S. Naval Medical Research Institute Detachment

**NMCP**  National Malaria Control Program

**PAHO** Pan American Health Organization

**USAID** United States Agency for International Development

**USG** United States Government

**WHO** World Health Organization

*** DISA** stands for “Dirección de Salud” (Health Directorate). The current term in use is DIRESA or “Dirección Regional de Salud” (Regional Directorate for Health).

| **Categories of Stakeholders** | **Stakeholders’ Affiliations** | **Roles** | **Level of Analysis**  **(H=High, M=Medium, L=Low)** | | |
| --- | --- | --- | --- | --- | --- |
|  |  |  | **Interest** | **Impact** | **Influence** |
| CDC | Foreign Government | Technical assistance: capacity building | H | L | M |
| DISAs | Sub-national Government | Health Authority, research, implementers | H | H | M |
| Health care workers | Government | Implementers | H | H | L |
| Health care workers | Private Sector | Implementers | H | H | L |
| INS | National Government | Research | H | M | H |
| Local communities | General Public | Beneficiaries | H | H | L |
| Media | Private Sector | Communication | H | L | M |
| Ministry of Health | National Government | Policy, Implementation | H | H | H |
| NAMRID | Foreign Government/Military | Funding, capacity building | H | L | M |
| NMCPs – Amazon Countries | Regional Governments | Regional policy | M | H | L |
| NMCP | National Government | Formulate policy, lead implementation | H | H | H |
| PAHO | Regional Bilateral Partner | Regional policy | H | M | M |
| Pharmaceutical companies | Private Sector | Beneficiary | H | H | L |
| Universities | Academia | Research | M | L | M |
| USAID | Development Assistance Agency | Funding, technical assistance | H | L | H |
| VIGIA Project | USG/GOP Partnership | Capacity building, promoting collaboration | H | H | H |
| WHO | Global Bilateral Partner | Global policy | M | L | M |
