# Supplementary material for: A retrospective analysis of the change in anti-malarial treatment policy: Peru
Source: Malar J. 2009 Apr 28;8:85. doi: 10.1186/1475-2875-8-85 (PMC2684118; doi:10.1186/1475-2875-8-85)
Supplement: Additional file 2 — Factors Impacting the Process of Changing National Anti-malarial Drug Policy in Peru. Factors that impacted the change in national anti-malarial drug policy are categorized by type and directionality (positive or negative). [file 1475-2875-8-85-S2.doc]

**Additional file 2: Factors Impacting the Process of Changing National Anti-malarial Drug Policy in Peru**

1. **Political:**
   1. Positive Factors:
      1. Strong political will of integrated group of researchers, clinicians and control personnel toward common goal of improving malaria control.
      2. Development of VIGIA Project that served as a bridge among stakeholders, donors and the Ministry of Health (MoH), including ties to higher levels of staff in MoH, other major divisions within the MoH and external partners.
      3. International endorsement for NMCP and, subsequently, the MoH.
      4. Media pressure during the El Niño epidemic, which caused the MoH to increase their attention on malaria.
      5. Development and dissemination of a policy document (“Politca Nacionals”) to communicate steps necessary for policy change, in advance of the actual change.
      6. National meeting for stakeholders and seminar for the community to lay the foundation for the needed change, utilizing evidence-based data.
      7. Vested interest in better malaria control by members of Parliament whose home districts were within malaria-endemic areas.
      8. Understood political base of power and used specific individuals and events to move agenda forward.
   2. Negative Factors:
      1. Instability of government, which slowed process and diminished the overall functioning of the health system organization.
2. **Socio-Cultural:**
   1. Positive Factors:
      1. Recognized need to capitalize on long-standing overlapping personal and professional social networks in which individuals trusted one another and shared a common vision.
      2. Stressed need for transparency in communications, which promoted better collaboration among stakeholders.
      3. Use of an individual stakeholder who had a personal relationship with the Minister of Health and, thus, could transmit pertinent information directly to the Minister.
      4. Perceived themselves (i.e., all those involved in process) as unified team, thus improving collaboration.
3. **Economic:**
   1. Positive Factors:
      1. Donor interest and support for national activities and international technical assistance when needed.
      2. Targeted donor funding to promote regional agenda for malaria control (“Amazon Malaria Initiative”).
      3. Use of economic data that discussed national implications of not changing malaria treatment policy, which garnered additional political support.
      4. Willingness of partners with differing objectives to share funds to address common research interest.
   2. Negative Factors:
      1. Initial lack of sufficient funding for research slowed process.
      2. Fears of political reprisal for spending funds during periods of government instability resulted in marked delays in procuring drugs for implementation.
4. **Scientific:**
   1. Positive Factors:
      1. Reported levels of increased drug resistance sparked interest in better documentation of problem.
      2. Global endorsement of improved methods for monitoring drug efficacy resulted in stronger, more credible data on which the argument for policy change could be based.
      3. Developed sufficient levels of efficacy and safety evidence-based data to promote the need for policy change.
5. **Environmental/Epidemiologic:**
   1. Positive Factors:
      1. Shifting pattern in specie predominance from *Plasmodium vivax* to *Plasmodium falciparum* raised level of concern.
      2. Epidemic situation resulted from El Niño weather event resulted in increased attention to malaria.
   2. Negative Factors:
      1. Different patterns of resistance within the country resulted in site-specific policy, which was more complex to implement than a single national policy.
6. **Organizational (focus on health systems):**
   1. Positive Factors:
      1. Inclusion of peripheral staff at early stages of the process promoted better collaboration, sense of ownership and strengthened local capacity in science and ability to negotiate with central level.
   2. Negative Factors:
      1. Instability of government resulted in loss of technical skills, institutional memory, and a marked decrease in human resources directed toward malaria control.
